# Phyllosphere Microbiome in Plant Health and Disease

**DOI:** 10.3390/plants12193481

**Published:** 2023-10-05

**Authors:** Surajit De Mandal, Junhyun Jeon

**Affiliations:** Department of Biotechnology, Yeungnam University, Gyeongsan 38541, Republic of Korea; mandal@yu.ac.kr

**Keywords:** phyllosphere microorganisms, plant defense, dysbiosis

## Abstract

The phyllosphere refers to the aboveground surface of plants colonized by diverse microorganisms. Microbes inhabiting this environment play an important role in enhancing the host’s genomic and metabolic capabilities, including defense against pathogens. Compared to the large volume of studies on rhizosphere microbiome for plant health and defense, our understanding of phyllosphere microbiome remains in its infancy. In this review, we aim to explore the mechanisms that govern the phyllosphere assembly and their function in host defence, as well as highlight the knowledge gaps. These efforts will help develop strategies to harness the phyllosphere microbiome toward sustainable crop production.

## 1. Introduction

The phyllosphere refers to the aboveground parts of plants and is considered a harsh habitat for bacterial colonization due to low water and nutrient availability, exposure to ultraviolet radiation, and day/night temperature fluctuations [1,2]. Despite such challenges, the phyllosphere is one of the most diverse ecosystems on earth, colonized by various microbes, including bacteria, fungi, viruses, algae, nematodes, and protozoa, collectively known as phyllosphere microorganisms [3]. It has been estimated that worldwide leaf areas occupy about 500 million km^2^, containing 10^6^–10^7^ bacterial cells per square centimeter [3,4].

The major sources of these microorganisms are soil or litter, seeds, and air, which are recruited either vertically or horizontally or through neighboring microbial reservoirs [5,6,7]. Furthermore, recent evidence suggests that leaf and root microbiota could arise from the same source, and these two microbial communities may interact through wind, insect, or plant vascular tissues [8,9,10,11,12]. These microorganisms either live on (epiphytes) or inside (endophytes) the aboveground plant tissues, having co-evolved with plants millions of years ago, and enhance the host’s genomic and metabolic capabilities, including pathogen defense and stress tolerance, the promotion of growth and reproduction, nutrient acquisition, and control of flowering phenology; ultimately contributing to plant health and performance [11,13,14,15]. Therefore, an in-depth understanding of phyllosphere microbial communities and their functional properties is of great significance for promoting plant health and agricultural production.

Over the course of evolution, plants have been equipped with various mechanisms that select specific microorganisms in the phyllosphere. An increasing number of studies have revealed that phyllosphere microbiomes share core bacterial phyla (such as Proteobacteria, Actinobacteria, and Bacteroidetes) or proteins across different plant hosts, which indicates the presence of a common mechanism for their adaptation or survival [15,16]. Deciphering the mechanisms by which the phyllosphere shapes and maintains a unique microbial community is challenging but critical for understanding plant–microbe interactions. However, to date, the rhizosphere microbiome has been a major focus for maintaining plant health and combating pathogens, while the phyllosphere microbiome remains largely elusive. In this review, we aim to explore the mechanisms of phyllosphere microbiome assembly and the functions of phyllosphere microbiome in host defence.

## 2. Mechanisms of Phyllosphere Microbiome Assembly

**Figure 1:**
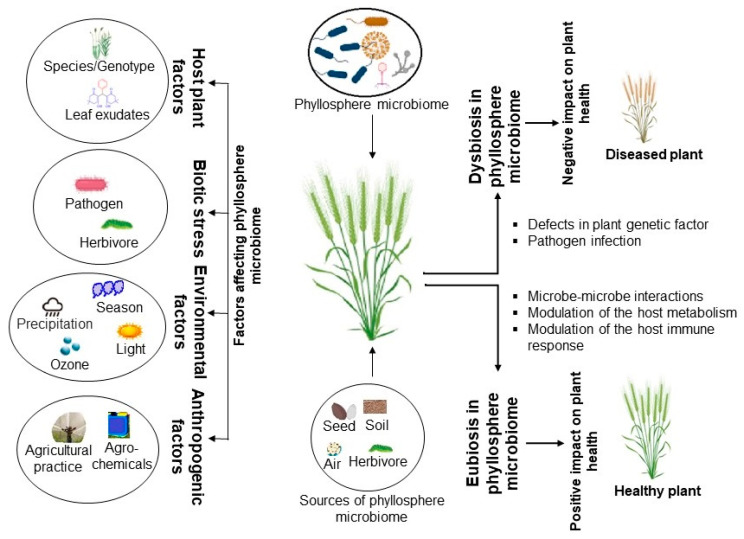
Major factors influencing microbiota assembly in the phyllosphere.

The unique diversity or community composition of phyllosphere microbes is important for beneficial plant traits, which underscores the importance of understanding the mechanisms that govern phyllosphere microbiome assemblages and how they positively impact the host plant. The host plant (genetics, hormones, metabolites), environmental factors, and functional activities of the microbial colonizers have been suggested to play key roles in microbial assembly and selection in the phyllosphere [17] (Figure 1).

### 2.1. Host Genotype

The genotype and phenotype of the host plant play a major role in structuring the phyllosphere microbial communities [18,19]. Plants with different genotypes develop different phenotypes, which in turn affect the phyllosphere microbiota [20]. In general, the leaf chemistry, morphology, and developmental stages significantly affect the phyllosphere microbiome. For instance, a mutation in cuticular wax biosynthesis affects the community composition of phyllosphere bacteria in *Arabidopsis thaliana* [21]. A genome-wide association study (GWAS) suggested that plant loci related to defense and cell wall integrity are likely involved in shaping the phyllosphere microbial community of *A. thaliana* [22]. Bodenhausen et al. (2014) studied plant host genotype-dependent community development using the SynCom approach, which revealed that cuticle synthesis and ethylene perception influence phyllosphere bacterial communities in *A. thaliana* [18]. Genes associated with defense, kinase-related activities, and cell wall integrity significantly impact the microbial community composition in the phyllosphere [23]. Similarly, mutations affecting cuticle synthesis alter the phyllosphere microbiome, which in turn helps the plant to resist the phytopathogen *Botrytis cinerea* [24]. These examples illustrate the role of plant genotype in shaping the phyllosphere microbial community. Apart from the genotype, plant growth/physiological stage or age affects phyllosphere microorganisms via the secretion of specific hormones and active substances [25,26].

### 2.2. Leaf Exudates

Beyond genetics, recent studies indicate that leaf exudates, including the primary and secondary metabolites, can regulate the phyllosphere microbiome. These compounds act as a critical nutrient source for phyllosphere inhabitants. For instance, the carbohydrates have been identified as a determining factors for growth and leaf colonization of *Pseudomonas syringae* pv. tomato and *Sphingomonas melonis* [27]. The phyllosphere inhabitant, *Methylobacterium*, utilizes methanol as a source of carbon and energy [28]. Several of these compounds have been reported to influence the developmental-stage-specific core microbiota, thereby regulating the phyllosphere microbiome. For instance, the tea alkaloid theophylline enriched in the early stages of development and protects young tissues, whereas the dominant catechin, epigallocatechin gallate, was identified in the late shoot stage and plays a major role in host defense against pathogen attack in the tea phyllosphere [29,30].

Moreover, certain metabolites such as coumarins, flavonoids, lignin precursors, quaternary ammonium salts, and terpenoids were enriched with the expansion of the lesion and had positive regulatory effects on *Rhynchogastremataceae*, *Golubeviaceae*, and *Actinomycetales*, playing a significant role in the assembly of phyllosphere microbial communities [31]. Plant hormones can also act as a selective force in the microbiome assembly process. For example, cytokinin has been reported to act as a signaling molecule that drives phyllosphere bacterial community assembly and increases disease resistance [32]. Graindorge et al. (2022) revealed that the metabolic status of the plant is vital for the recruitment of *Streptomyces* into the microbiota [33]. This evidence indicates that the plant could favor specific microorganisms by releasing specific volatile compounds. Therefore, future studies should focus on identifying these compounds or their biosynthetic pathways that could lead to artificial regulation of phyllosphere microbial composition to improve plant health and crop productivity.

### 2.3. Environmental Factors

Environmental factors, such as geographic location, climatic conditions, temperature, moisture, CO_2_, etc., strongly affect the phyllosphere microbial community [12,34]. For instance, temperature and CO_2_ have been reported to affect the molecular pathways and significantly affect the diversity and community composition of phyllosphere microorganisms [35,36,37]. These impacts make the phyllosphere an important habitat for thermophilic microorganisms [38]. In addition, precipitation serves as a major factor that shapes the distributions of phyllosphere microorganisms [39]. It has been reported that light can affect the interaction between the phyllosphere microbiota and plant hosts [3,40]. Geographic location and season have been shown to influence the phyllosphere microbial communities [41,42,43]. A recent study illustrated that O_3_ and water deficit stress on the phyllosphere microbial community could reduce alpha diversity and the abundance of Betaproteobacteria and an increase in Gammaproteobacteria abundance, indicating that these microbial shifts or the dysbiosis-related biosignature can be used to access poplar disease risk [44].

### 2.4. Anthropogenic Factors

Several anthropogenic factors, such as intensive agriculture, deforestation, urbanization, and pollution [34,45], could interfere with the phyllosphere microbial communities. Compared to the soil, the functional diversity of phyllosphere microbes is more sensitive to these disturbances; therefore, any small changes in the phyllosphere microbes can drastically impact the ecosystem [46]. Increasing evidence has shown that the application of excess fertilizer negatively impacts the diversity and functional attributes of the phyllosphere microbiomes. For instance, excessive use of nitrogen fertilizer results in an increased abundance of fungal plant pathogens in the phyllosphere [47]. Berg and Koskella (2018) showed that fertilization makes a plant more vulnerable to pathogen attacks [48]. A recent study revealed that the application of tebuconazole altered the fungal microbiome by decreasing the abundant fungal members, including the potentially beneficial endophytic fungi, while NPA (Neutralized Phosphorous Acid) and sulfur had minimal impacts on the phyllosphere fungal microbiome [49]. Urbanization leads to an increase in human activities and has a strong effect on the phyllosphere microbiota [50,51,52]. Analysis of phyllosphere microbial communities of seven tree species in Montreal, Canada, showed that alpha diversity increased with urban intensification [50]. Another study by Espenshade et al. (2019) also observed the impact of urbanization (i.e., traffic patterns and urban density) on the phyllosphere microbial community, which was correlated with black carbon and ultrafine particulate matter on tree leaves [51]. However, further research is needed to determine how these taxonomic changes affect the functional characteristics of the phyllosphere microbiota.

### 2.5. Microbe–Microbe Interactions and Herbivores Impact

Members of the plant microbiota are involved in a wide range of interactions with each other (intraspecific or interspecific) that affect the community structure and functional properties of the microbiome. These interactions are either cooperative (mutualism and commensal), parasitic, or competitive (antibiosis, competition for nutrients or space) and can be formed within or between bacteria–bacteria, fungus–fungus, bacteria–fungus, bacteria–virus, etc. [2,53]. Herbivorous insects can alter the phyllosphere’s microbial community and make the host more susceptible to pathogens. For example, herbivores significantly increase the abundance of bacterial groups in the leaves of bittercress [54]. In addition, a higher bacterial diversity was reported in leaves damaged by lepidopteran larvae [55]. Possible mechanisms behind this could be the alteration of the plant defense response, release of nutrients from damaged tissues, or direct addition of microbes from herbivores to plant tissues [54,56].

The phyllosphere microbial community assembly is also influenced by the priority effects, i.e., the order and timing of the arrival of species during community assembly [57,58]. Early leaf colonizers gain a numerical advantage to colonize and establish themselves, which reduces the colonization success of the later species. Using nectar-dwelling microorganisms as a model system, Tucker and Fukami (2014) revealed that priority effects result in the exclusion of late-arriving species if temperature is held constant while temperature variability prevents their extinction [57]. Another study by Carlström et al. (2019) showed that microbial community assembly in the *Arabidopsis* phyllosphere was subject to priority effects, and keystone taxa such as *Sphingomonas*, *Rhizobium*, *Microbacterium*, and *Rhodococcus* play a vital role in affecting the microbial community structure [58]. It has also been reported that the selection of a stable microbiome that is well adapted to a plant is possible through successive passaging approaches [59]. Therefore, priority effects not only impact the phyllosphere microbial community structure but also play a significant role in its functional potential.

## 3. Role of the Phyllosphere Microbiome in Plant Defence

Phyllosphere harbors diverse microbial taxa that can have positive, negative, or neutral effects on the host plant. Here, we present their role in host defense that occurs through multiple mechanisms, such as microbe–microbe interaction, modulation of host metabolism, or activation of plant immunity (Table 1).

### 3.1. Microbe–Microbe Interactions

Microbe–microbe interactions have a strong effect on the functional diversity of the phyllosphere microbiome, which affects host phenotype. Direct competition between pathogenic and non-pathogenic microbes in the phyllosphere for space and nutrients plays an important role in the biocontrol of pathogens. For instance, the *Sphingomonas* strains could limit the plant pathogen *P. syringae* by directly competing for glucose, fructose, and sucrose [72]. It has also been suggested that the presence of diverse microbial communities may increase competition with pathogens for shared resources, such as nutrients or space; therefore, higher microbial diversity in the phyllosphere may help protect plants from pathogenic infection [75,76].

Plant-associated microbes, including phyllosphere microorganisms, are known to produce various secondary metabolites that play an important ecological role in microbial communities by promoting and inhibiting microbial activities. For example, *Enterobacter aerogenes*, the endophytic bacterium of maize, has been reported to produce 2,3-butanediol, which increases host resistance against phytopathogens [77]. Helfrich et al. (2018) identified several biosynthetic gene clusters (BGCs) that can synthesize novel natural products with antimicrobial potential [78]. Similarly, 3-methylbutan-1-ol produced by the tomato phyllobacterium, *Enterobacter cloacae* TR1, suppresses the growth of *B. cinerea* [68].

In addition to directly suppressing invading pathogens, several signaling molecules have been identified in the phyllosphere microbes that interfere with pathogen virulence. For example, *Microbacterium testaceum* isolated from the phyllosphere of *Solanum tuberosum* showed putative AHL-lactonase activity and can inactivate both short- and long-chain AHLs, thereby inhibiting bacterial infection by the *Pectobacterium carotovorum* subsp. Carotovorum [73]. Lactonase-producing *Acinetobacter* sp., *Bacillus* sp., *Lysinibacillus* sp., *Myroides* sp., *Pseudomonas* sp., and *Serratia* sp. from the tobacco phyllosphere have been reported for AHL degrading activity and may be further developed for effective biocontrol agents against phytopathogens [79]. A recent study by Zhang et al. (2023) demonstrated that a functional quorum sensing (QS) circuit is essential to establish colonies in the phyllosphere and suppress pathogens by *Rhodopseudomonas palustris* GJ-22 [80]. However, so far, only a limited number of phyllobacteria have been investigated for their ability to produce antimicrobial compounds, including various signaling metabolites. Therefore, future studies should focus on identifying and characterizing these molecules that may lead to the discovery of novel antimicrobials from the phyllosphere microbiota. The increasing availability of genomic data together with advances in metabolomics tools, will play a key role in these investigations.

### 3.2. Modulation of the Host Metabolism

Phyllosphere microbes can modulate host gene expression to assist pathogen defense. They can alter the emission of plant volatile organic compounds (VOC) by inducing plant defense responses or disrupting normal metabolism. Liu et al. (2023) reported that the rice panicle microbiome regulates host metabolism to confer resistance to rice against the pathogen *Ustilaginoidea virens*, which causes false-smut disease. They revealed that the panicle microbial community of disease-suppressing plants targets aminotransferases and modulates branched-chain amino acid (BCAA) levels in the panicle, leading to plant defense against the pathogen. This evidence suggests that the application of microbial agents that elicit BCAA or exogenous BCAA application could serve as an alternative to chemical fungicides [62]. Another study by Gargallo-Garriga et al. (2016) demonstrated that suppression of phyllosphere microbial communities by antibiotic fumigation reduces the concentration of acetyl-CoA, citraconic acid, isoleucine, and other secondary compounds, including phenols and terpenes in *Sambucus nigra*. This suggests that phyllosphere microbes aid in the plant’s ability to produce various compounds that support plant health and productivity [81].

### 3.3. Modulation of the Host Immune Response

Plants have evolved with a robust defense system to target potential pathogens either by recognizing microbe or pathogen-associated molecular patterns (MAMPs or PAMPs) via pattern recognition receptors (PRRs) resulting in MAMP- or PAMP-triggered immunity (MTI or PTI); or through effector-triggered immunity (ETI) involving recognition of effectors via nucleotide-binding leucine-rich receptor (NLR) [82]. This MTI/ETI immune defense system is based on the bidirectional interaction between plants and pathogens. However, growing evidence suggests that non-pathogenic microbiota can also trigger the immune system and thereby help the plant to suppress invading pathogens. For example, *Sphingomonas* from *Arabidopsis* leaves could alter the expression of 400 genes, including signaling and defense responses that promote immunity against the pathogen *Pseudomonas* [10]. It has also been reported that *Rhodopseudomonas palustris* GJ-22 can be used to induce resistance in tobacco plants against tobacco mosaic virus (TMV) by spraying it on the leaves [83]. These beneficial microbes contain MAMPs that can be recognized by PRRs, so it is important to know how plants distinguish between beneficial and pathogenic microbes. In a study by Bozsoki et al. (2020), it was reported that legume plant use LysM1 motif of LysM-RLK for the recognition of symbiotic partners and discrimination of pathogenic microbes [84]. Another study identified a set of 24 immune-related genes in *A. thaliana* by analyzing the transcriptional response against bacterial inoculants derived from the *Arabidopsis* phyllosphere (At-LSPHERE). These genes are termed general non-self-response (GNSR) and play a significant role in distinguishing pathogenic from non-pathogenic microbes [85]. However, it is important to note that these molecular mechanisms can vary greatly depending on plant species, microbial types, and specific environmental conditions. Investigations are ongoing in this domain, and our understanding of these interaction mechanisms continues to evolve.

## 4. Future Research Topics

With the advancement of high-throughput molecular technologies and the development of novel approaches, such as the development of artificial communities (SynCom), computational multiomics enables us to decipher the taxonomic and functional properties of the phyllosphere microbiome in diverse host plants. Increased understanding of host-microbe interaction dynamics in the phyllosphere has further helped in developing strategies to improve plant phenotypes for agricultural productivity. However, much work remains to be carried out to address several challenges and unexplored aspects in the phyllosphere. In this context, we highlight important questions that, in our view, require future attention to fill the knowledge gaps in phyllosphere research.

(I) *How does a plant regulate phyllosphere bacteriophage communities?*

Phages are known to infect microbial cells and maintain their proper balance in the ecosystem and have been successfully used to control various pathogens [86,87]. Although their effects on the soil and marine microbiomes have already been extensively studied, such research on phyllosphere microorganisms is limited. Limited studies illustrate their potential role in disease control in the phyllosphere, but how plants regulate phyllosphere bacteriophage communities is lacking in the literature [88]. It is important to note that methodological constraints associated with sampling restrict the analysis of the actual phage population. Therefore, the mechanisms of plant-phage and bacterium-phage interactions in the phyllosphere are largely unexplored and should be elucidated in future studies. Bacteriophage-mediated phyllo-microbiome engineering can be adapted to modify microbial communities or remove pathogenic microbial members that will improve plant defense and productivity [86,89].

(II) *Does the “cry for help” strategy apply in the phyllosphere?*

Increasing evidence suggests that plants produce various chemical stimuli to recruit beneficial microbes or change their microbial communities in response to pathogen infection. This phenomenon is called the “cry for help” strategy, where plants actively cooperate with the microorganisms to cope with the disease. Despite considerable research on the “cry for help” strategy in the rhizosphere [90,91,92], evidence for this defense strategy has been largely overlooked in the phyllosphere. Recent studies have shown that plants can recruit microbial members in the phyllosphere to fight against pathogens [93,94,95]. For instance, infection by the fungal pathogen *Diaporthe citri*, in *Citrus unshiu* leaves leads to an intense microbial network and the emergence of large numbers of new microbes that support the “cry for help” strategy of the plant phyllosphere. The joint contribution of the native microbes and recruited new microbes leads to changes in the functional dynamics of the entire microbial community, such as the enrichment of iron competition and potential antifungal properties, ultimately benefiting the host [95]. This evidence indicates that during pathogen attack, host plants undergo disease-suppressive microbiome assembly processes in the phyllosphere. However, the molecular mechanisms by which hosts alter their phyllosphere microbiota are largely unknown and thus need future investigation.

(III) *How does a plant maintain the phyllosphere microbial homeostasis?*

Growing evidence indicates that the host and its microbiome have evolved with multiple strategies to cooperate bidirectionally, which benefits the host’s health. Moreover, the selection of the right microbiome and maintaining homeostasis is vital for plant health. However, the host factor associated with the homeostasis of the phyllosphere microbiome is largely unknown. Emerging studies show that PTI plays a critical role in modulating microbiota homeostasis in plants. For example, Chen et al. revealed that *Arabidopsis* quadruple mutants (*min7 fls2 efr cerk1* (*mfec*)) which are defective in the PTI and MIN7 vesicle trafficking pathway, and a *constitutively activated cell death1* (*cad1*) mutant, have altered leaf endophytic bacterial diversity [96]. Another study showed that *Arabidopsis* mutants defective in NADPH (Nicotinamide adenine dinucleotide phosphate) oxidase RBOHD (respiratory burst oxidase homolog protein D) have altered phyllosphere microbiota [97]. These studies have revealed that several plant genetic factors, including PRR signaling, MIN7, cad1, NADPH oxidases, etc., play an essential role in leaf microbiota homeostasis. Future research should explore other genetic factors that regulate the phyllosphere microbiome and how we can harness these factors to engineer plants that can help to colonize beneficial microbes.

(IV) *How does a disturbed phyllosphere microbiome affect the host plant?*

Under stressed conditions, the normal microbiome’s homeostasis is often disrupted (dysbiosis), and the host becomes more susceptible to harmful microbial invaders, resulting in a negative impact on the plant. This phenomenon is widely studied in humans and linked with the development of important diseases. In general, dysbiosis is defined as the disturbance of the microbiome due to the imbalance of the gut microbial communities or the imbalance between the beneficial and harmful microorganisms due to the loss or gain of microbial members or changes in their abundance [98]. Defects in plant genetic networks often result in the formation of phyllosphere dysbiosis and the development of various plant diseases or disease symptoms. For example, Chen et al. (2020) reported that microbiome dysbiosis in the phyllosphere resulted in decreased bacterial richness and conversion of Firmicutes-rich communities into Proteobacteria-rich communities and the occurrence of disease symptoms (leaf chlorosis and necrosis) in the *Arabidopsis*. Therefore, phyllosphere dysbiosis could lead to the loss or decrease of beneficial microbes associated with pathogen suppression, leading to the enrichment of opportunistic pathogens and ultimately reducing disease resistance in the plant [96]. In general, opportunistic pathogens contain potential virulence functions and are generally harmless to host plants but can cause disease under specific conditions such as microbial dysbiosis. For instance, the rbohD knockout in plants leads to the proliferation of opportunistic pathogens in the phyllosphere [97]. The opportunistic strain *Xanthomonas* Leaf131 and Leaf148 has been reported to secrete cell-wall-degrading enzymes through the T2SS, leading to the degradation of surrounding tissue, which promotes their growth during infection [99]. Therefore, under conditions of dysbiosis, phyllosphere commensals could turn pathogenic and cause disease by secreting important virulence factors.

Pathogens that invade the plant often cause dysbiosis in the phyllosphere microbiome either by targeting the plant immune system, which indirectly affects other microbes, or by directly targeting microbial communities by releasing proteins and peptides with antimicrobial activities. They often target keystone microbial species that facilitate the formation and integrity of a community, leading to microbial network collapse and negative effects on the host plant [41]. For example, the fungal pathogen *Zymoseptoria tritici* can suppress the immune system of wheat, thereby altering the leaf microbial community and making the host more vulnerable to further infection [100]. Dysbiosis due to infection or other factors significantly impacts the diversity and community of the phyllosphere microorganisms. For example, a study on *Cucumis sativus* and *Euonymus japonicus* revealed that powdery mildew infection results in greater diversity and richness of the epiphytic bacterial community [101]. However, the fungal diversity in the tobacco phyllosphere decreased with the increasing leaf spot disease caused by *Didymella segeticola* [102]. Plants recruit more bacteria to prevent pathogen invasion, which could lead to increased bacterial diversity, whereas fungal pathogens compete for host nutrition, which may suppress the growth and reproduction of other fungi, thereby decreasing their diversity [103]. Therefore, microbial community composition in the phyllosphere changes over the development of the disease, ultimately affecting the stability and function of the microbial network in the phyllosphere [104]. Although several of these observations have been reported, more research is needed to address the new challenges associated with phyllosphere dysbiosis and to develop strategies that can prevent dysbiosis and support plant health and productivity.

(V) *What are the major methodological constraints for analyzing the phyllosphere microbiome?*

The study of the phyllosphere microbial community and its functional attributes is often hampered due to multiple limitations. For instance, the low abundance of phyllosphere microbes makes it difficult to extract their genomic materials from the leaf. The available extraction methods of leaf microbial DNA/RNA allow the extraction of microbial and host genomes, making subsequent metagenomic analysis difficult. A recent report by Nobori et al. (2020) suggests that centrifugation can be applied to enrich microbial fractions from plant material using centrifugation for next-generation sequencing [105]. The phyllosphere is rich in chlorophyll, and most of the amplicon metagenomics data generated from the phyllosphere are contaminated with the host sequences associated with chloroplast, ribosome, and mitochondria, thus making it difficult to recover the actual genomic information from the sequences. In general, these host-associated reads can be avoided using primer choice or removed during the data processing steps [106]. Recently, scientists have developed a newer method of microbiome analysis using peptide nucleic acids (PNAs) clamping approach and successfully used to suppress the amplification of plant contaminants such as plastid and mitochondrial sequences during PCR amplification [107,108,109]. Another study developed a CRISPR/Cas-based system that can cleave and remove the host rRNA amplicons [110]. Although some progress has been made, further research is needed to optimize the methodologies with advanced techniques and bioinformatics tools to enhance our understanding of chloroplast-rich phyllosphere microbial communities and their functional attributes.

## 5. Conclusions

The phyllosphere is a complex and variable environment colonized by various microorganisms that have various roles in plant growth and productivity. Recent advances in omics tools and the introduction of the SynCom application have enabled researchers to gain a deeper understanding of these communities and their intricate associations with host function. In this review paper, we have described our current understanding of phyllosphere biology and highlighted the assembly mechanisms and functions of the phyllosphere microbiome. Several challenges and unexplored areas such as the mechanism between plant–phage or bacteria–phage interactions, disease-suppressive microbiome assembly mechanisms under pathogen attack, strategies that can prevent phyllosphere dysbiosis and support plant health, etc., have also been discussed. Addressing these challenges will help to develop novel approaches for utilizing the phyllosphere microbiome in sustainable crop production.

## Figures and Tables

**Table 1:** Phyllosphere microbes and their role in plant disease resistance.

Plant	Pathogen	Phyllo Microbe	Mechanisms	Reference
*Oryza sativa*	*Pyricularia oryzae*	*Actinomycetes*	Produce bioactive compounds	[60]
	*Magnaporthe oryzae*	*Aspergillus cvjetkovicii*	Produces 2(3H)-benzofuranone and azuline, which suppress mycelial growth and appressorium formation	[61]
	*Ustilaginoidea virens*	Panicle microbes	Modulates the levels of branched-chain amino acids	[62]
*Zea mays*	*Exserohilum turcicum*	*Enterococcus*, *Corynebacterium*, *Pantoea* and *Bacillus*	Unknown mechanism	[63]
	*Bacillus subtilis* strain DZSY21	*Bipolaris maydis*	Reduce infection, possibly using antifungal lipopeptides and induced systemic response	[64]
*Triticum aestivum*	*Fusarium gramineareum*	*Pseudomonas piscium*	Compound secreted by the bacteria (phenazine-1-carboxamide) deregulates histone acetylation and suppress growth, virulence, and mycotoxin biosynthesis.	[65]
*Solanum lycopersicum*	*Pseudomonas syringae* pv. tomato and *Alternaria solani*	*Rhizobium* sp. and *Bacillus subtilis*	Produce protease and cellulase and induce salicylic acid (SA) immune response pathway	[66]
	*Botrytis cinerea*	*Bacillus* sp.	Produce lipopeptides antibiotics belonging to fengycin, surfactin, iturina and bacillomycin D	[67]
	*Botrytis cinerea*	*Enterobacter cloacae* TR1	Produces antifungal volatile compound 3-methylbutan-1-ol	[68]
*Nicotiana tabacum*	*Pseudomonas syringae* pv. *tabaci*	*Stenotrophomonas*, *Achromobacter*, *Enterobacter*, *Ochrobactrum*, *Pseudomonas*, *Bacillus*, *Alcaligenes*, *Pseudochrobactrum* and *Achromobacte*	Increases the complexity of microbial networks in the phyllosphere and establishes a “spatial repellent barrier” against invading pathogens	[69]
*Citrus limon*	*Xanthomonas citri* ssp. *Citri*	*Pseudomonas protegens* CS1	Inhibit pathogen by producing siderophore pyochelin	[70]
*Arabidopsis thaliana*	*Albugo laibachii*	*Moesziomyces bullatus ex Albugo*	GH25 hydrolase secreted by the commensal play a major role in pathogen defence	[71]
	*Sphingomonas melonis* Fr1	*Pseudomonas syringae* DC3000	Activates defence genes to promote immunity against pathogen	[10]
	*Pseudomonas syringae* pv. tomato DC3000	*Sphingomonas*	Substrate competition plays a role in plant protection	[72]
*Solanum tuberosum*	*Microbacterium testaceum*	*Pectobacterium carotovorum*	Interfere with the N-acyl-homoserine lactone (AHL)-based quorum-sensing of the pathgoen	[73]
*Brassica rapa*	*Alternaria brassicicola* ABA-31	*Bacillus subtilis* PMB102	Production of antifungal metabolites	[74]

## Data Availability

Not applicable.
